# Fulminant Clostridioides difficile Colitis With SARS-CoV-2 Infection

**DOI:** 10.7759/cureus.38401

**Published:** 2023-05-01

**Authors:** Sanchit Duhan, Bijeta Keisham, Ahlaa Salim

**Affiliations:** 1 Internal Medicine, Sinai Hospital of Baltimore, Baltimore, USA

**Keywords:** covid-19, infectious colitis, sars-cov-2 infection, clostridioides difficile colitis, clostridioides difficile infection

## Abstract

*Clostridioides difficile* (*C. difficile*)and coronavirus disease 2019 (COVID-19) infections can have overlapping symptoms. Recently, the association and outcomes of coinfection have been studied. We present the case of an 83-year-old lady with Parkinson’s disease (PD) who was admitted with pneumonia secondary to severe acute respiratory syndrome coronavirus-2 (SARS-CoV-2) infection. She was treated with empiric antibiotics ampicillin-sulbactam and azithromycin, along with antiviral therapy remdesivir and baricitinib, and dexamethasone. The patient developed severe *C. difficile* infection with a leukemoid reaction. She was treated with intravenous metronidazole and oral vancomycin without any improvement. Before she could receive a fecal microbiota transplant, her infection progressed to fulminant colitis, and she required emergent surgery. The patient developed several complications post-surgery and succumbed to the severe illness. Our patient’s multiple comorbidities and an underlying COVID-19 infection predisposed her to severe illness. This case emphasizes the long-standing discussion on antibiotic stewardship and encourages a debate on the role of immunosuppressant antiviral medications and underlying PD in predisposing patients to a severe *C. difficile* infection.

## Introduction

*Clostridioides difficile* (*C. difficile*) infection (CDI) was the most common infection in hospitals in the United States of America (USA) before the coronavirus disease 2019 (COVID-19) pandemic [1]. During the COVID-19 pandemic, there was a significant increase in the use of certain antibiotics, including ceftriaxone, a third-generation cephalosporin [2]. Cephalosporin use has traditionally been linked to an increased risk of CDI [3]. However, severe acute respiratory syndrome coronavirus-2 (SARS‑CoV‑2) infection can cause gut dysbiosis and deplete symbionts [4]. Hence, patients often develop gastrointestinal symptoms, including nausea, vomiting, and diarrhea [5]. This overlap of symptomatology could explain the paradoxical decline trend of *C. difficile* testing and unchanged rates of CDI observed during the pandemic despite the increased rate of cephalosporin use. Another explanation could be improved infection control measures during the pandemic [6]. CDI and SARS-CoV-2 coinfection rates vary from 0.4% to 10% in different studies [7]. There are reports of higher mortality rates in patients with CDI and SARS-CoV-2 coinfection, but the data regarding the outcomes and severity is minimal [8]. There have been sporadic reports of fulminant *C. difficile* colitis in the backdrop of SARS-CoV-2 infection [9].

## Case presentation

An 83-year-old lady with Parkinson's disease (PD), who was unvaccinated for SARS-CoV-2, presented with shortness of breath for two days. She denied any abdominal pain and had regular bowel movements. The patient was recovering in a rehabilitation facility after a vertebral fracture post-mechanical fall. Her baseline mental status was limited in the setting of advanced PD and was only orientated to place and person.

**Figure 1:**
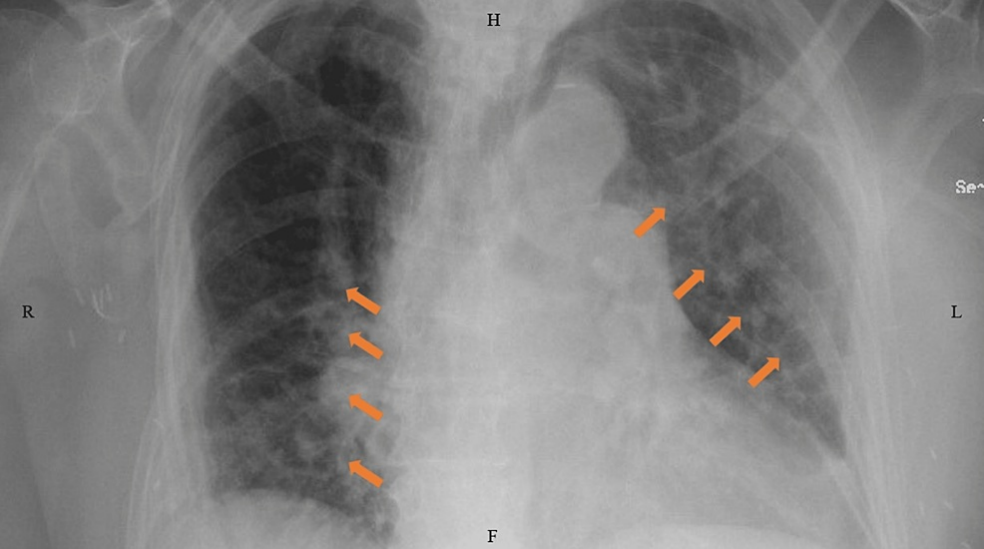
Chest x-ray showing interstitial edema (arrows). R: right; H: head end; L: left; F: foot end

Upon presentation, her mentation was at baseline, and her examination was only remarkable for bilateral bibasilar crackles on chest auscultation. Her abdomen was soft, non-tender, non-distended, and had normal bowel sounds. She required 4 L of oxygen support with a nasal cannula. Initial laboratory workup showed an elevated white blood cell (WBC) count of ~14,000 cells/mm^3^ (normal range: 4,500-11,000 cells/mm^3^). A chest x-ray showed signs of interstitial edema (Figure 1).

**Figure 2:**
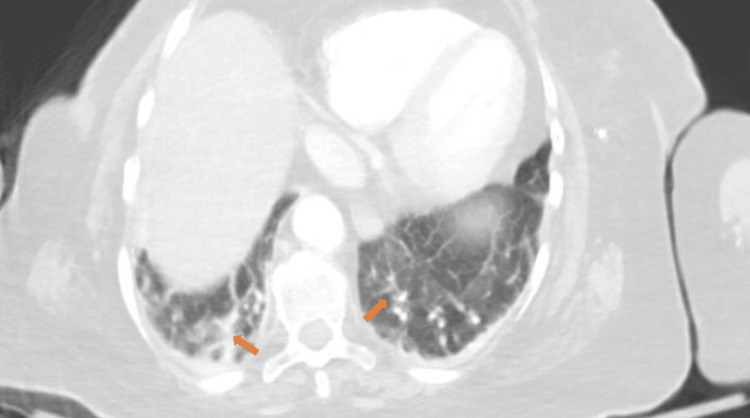
Bilateral pulmonary scarring and atelectasis on computed tomography (CT) scan (arrows).

**Figure 3:**
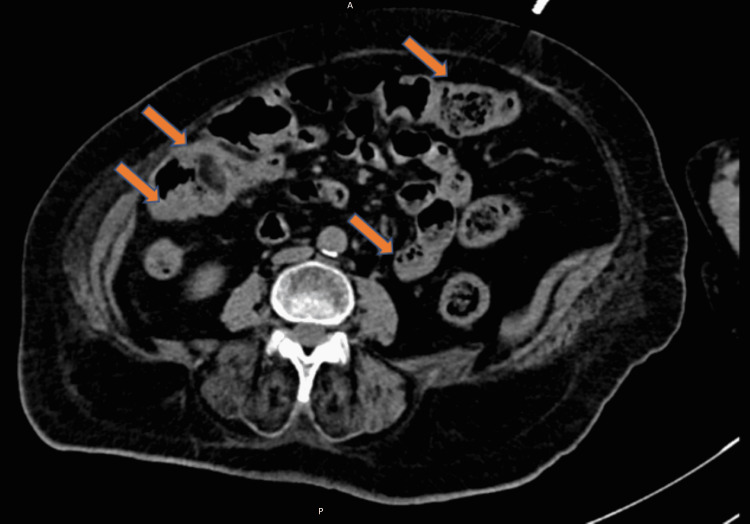
Computed tomography (CT) scan showing C. difficile colitis (arrows). A: anterior; P: posterior

A computed tomography (CT) scan of the chest revealed mild atelectasis and scarring bilaterally, most prominent at lung bases, but no definitive consolidation and embolus were seen (Figure 2). Given the patient's advanced dementia, risk of aspiration, and atypical x-ray findings, she was empirically treated with ampicillin-sulbactam and azithromycin. On the second day, the patient was found positive for SARS-CoV-2 on a reverse transcription-polymerase chain reaction (RT-PCR) test. Her c-reactive protein (CRP) was elevated to 167 mg/dL (normal range: less than 0.3 mg/dL). Treatment with remdesivir, baricitinib, and dexamethasone was initiated along with the antibiotics, given the suspicion of bacterial superinfection over viral pneumonia. Her bowel movements were regular and well-formed. By day five, the patient's oxygen requirement improved to requiring one liter of oxygen support. However, a rising trend was seen in the white count, initially attributed to steroid treatment. But given a further increase in WBC count to ~26,000 cells/mm^3^ with neutrophilic predominance on day six, her antibiotics were switched to vancomycin and piperacillin-tazobactam for broader coverage. The patient developed severe diarrhea (more than five bowel movements per day) and worsening abdominal pain in the next two days. On day eight, a polymerase chain reaction (PCR) assay for *C. difficile* toxin was positive, and a CT scan of the abdomen showed colitis with rectal thickening (Figure 3).

The same day, baricitinib was discontinued after seven days of therapy. Given the suspicion of fulminant *C. difficile *colitis, treatment with oral vancomycin and intravenous metronidazole was initiated. The plan for a fecal microbiota transplant was underway. However, the patient's mental status worsened, and she remained somnolent through most days. Her abdomen was distended and diffusely tender but without signs of peritoneal involvement (no rigidity or rebound tenderness). She had mean arterial pressures ranging from 65 to 70 mmHg (normal range: 70-100 mmHg) and a worsening leukocytosis (WBC>50,000 cells/mm^3^ on day nine) (Table 1).

**Table 1:** Relevant laboratory parameters over the course of hospitalization.

Days since admission	Laboratory parameter	Patient’s value	Normal range
Day 1	White blood cell (WBC) count	~14,000 cells/mm^3^	4,500-11,000 cells/mm^3^
Day 2	C-reactive protein	167 mg/dL	<0.3 mg/dL
Day 6	WBC count	~26,000 cells/mm^3^	4,500-11,000 cells/mm^3^
Day 9	WBC count	>50,000 cells/mm^3^	4,500-11,000 cells/mm^3^
Day 22	Lactic acid	10.7 mmol/L	0.5-1 mmol/L

Her ATLAS score was 7, indicating a 12.5% mortality risk [10]. The surgery team was consulted, and the patient was taken for an emergent (level 1A procedure) total abdominal colectomy with end ileostomy. Intra-operatively dilated small and large bowels were seen filled with dark-brown fluid. However, the patient could not be extubated post-procedure and developed septic shock requiring intravenous vasopressors. There was a suspicion of mucus plugs obstructing larger airways. She was transferred to the intensive care unit (ICU). Between days 11 and 22, the patient was treated with rectal vancomycin, oral vancomycin, and intravenous metronidazole. But her ICU stay was complicated with worsening sepsis leading to acalculous cholecystitis, diffuse anasarca, atrial fibrillation with a rapid ventricular response, deranged liver functions, and severe lactic acidosis (peak: 10.7 mmol/L, normal range: 0.5-1 mmol/L). Despite aggressive resuscitative efforts, she passed away on day 22 of hospitalization.

## Discussion

CDI manifestation varies from asymptomatic cases and mild diarrhea to severe fulminant colitis, pseudomembranous colitis, toxic megacolon, and death. CDI and severe COVID-19 infections lead to the production of similar cytokines in the body. Especially the Th17 cytokines IL-6 and IL-23 are associated with disease severity in CDI and COVID-19 both. COVID-19 infection can cause alteration of fecal microbiota leading to depletion of beneficial commensals like *Bacteroides dorei* and *Bacteroides thetaiotaomicron*, which can predispose the patients to a higher prevalence of *C. difficile* due to dysbiosis. Other possible predisposing factors include broad-spectrum antibiotics use in COVID-19 infection. Since the symptomatology of COVID-19 pneumonia resembles bacterial pneumonia, patients are often treated with antibiotics even though the rate of bacterial coinfection at admission is meager. The commonly associated antibiotics include clindamycin, cephalosporins, penicillin, and fluoroquinolones. The World Health Organization (WHO) has well-defined recommendations regarding the use of antibiotics in hospitalized patients with COVID-19 with bacterial superinfection. Still, the overuse of broad-spectrum antibiotics remains a significant issue. Almost 75% of patients from a long-term facility generally receive at least one course of antibiotics [11].

A study assessing the patients on antibiotics with *C. difficile* and COVID-19 co-occurrence by Sandhu et al. reported very high ATLAS scores in this subgroup with a 44% mortality [8]. Another more extensive retrospective study by Maslennikov et al. showed a higher risk of co-occurrence in women and increased mortality risk by 2.6 times [7]. The antibiotics associated with CDI in COVID-19 patients were levofloxacin, amoxicillin/clavulanate, and glucocorticoids. No increased risk was seen with a combination of ceftriaxone and azithromycin without glucocorticoids and other antibiotics [7].

CDI in the elderly has a higher risk of mortality and functional decline. Especially patients suffering from dementia or delirium are more likely to have fatal outcomes during hospitalization [12]. The recent literature suggests the role of fecal microbiota transplant in PD and delirium treatment, as PD is associated with alteration in the gut microbiome and gastrointestinal dysfunction. However, there is only anecdotal evidence in the form of case reports [13,14]. Our patient could not receive the microbiota transplant, given the rapid progression. But we believe it is worth performing a prospective study to find any benefit of using fecal microbiota transplant in PD patients with CDI to help overcome delirium and improve the chances of recovery.

Another factor worth mentioning is baricitinib, a Janus kinase (JAK) inhibitor that inhibits type I/II cytokine receptors and causes immunosuppression which can predispose to secondary bacterial infection [15]. There is a lack of data regarding any association of baricitinib with CDI, but we wonder if its use predisposed our patient to a severe CDI. The association and effect of baricitinib on CDI disease course and progression should be studied in randomized controlled trials to give us a better perspective.

Multiple factors that played a role in the adverse outcome of our patients need to be highlighted. The key elements include - (1) co-occurrence of COVID-19 infection and CDI leading to a higher mortality risk, (2) use of antibiotics for bacterial superinfection, (3) older age, and (4) underlying dementia.

## Conclusions

COVID-19 infection can predispose patients to CDI at a molecular level by causing dysbiosis, but the overuse of antibiotics in COVID-19 conditions remains a significant risk factor. Clinicians should be cautious and monitor patients closely who are being treated with baricitinib for COVID-19 and receiving empiric treatment for bacterial pneumonia. CDI may be fatal in these cases. Older women admitted for COVID-19 infections are at increased risk of severe CDI and mortality. Some antibiotics have a more predisposition than others, and more focused research in antibiotics use for bacterial superinfection must be investigated. The role of immunosuppressant medications used for COVID-19 treatment in the exacerbation of CDI is unclear. The emerging evidence of the usefulness of fecal microbiota transplants in patients with dementia needs to be studied in prospective trials.

## References

[1] Saha S, Khanna S (2019). Management of Clostridioides difficile colitis: insights for the gastroenterologist. Therap Adv Gastroenterol.

[2] Rose AN, Baggs J, Wolford H (2021). Trends in antibiotic use in United States hospitals during the coronavirus disease 2019 pandemic. Open Forum Infect Dis.

[3] Mylonakis E, Ryan ET, Calderwood SB (2001). Clostridium difficile-associated diarrhea: a review. Arch Intern Med.

[4] Zuo T, Zhang F, Lui GC (2020). Alterations in gut microbiota of patients with COVID-19 during time of hospitalization. Gastroenterology.

[5] Tariq R, Saha S, Furqan F, Hassett L, Pardi D, Khanna S (2020). Prevalence and mortality of COVID-19 patients with gastrointestinal symptoms: a systematic review and meta-analysis. Mayo Clin Proc.

[6] Khanna S, Kraft CS (2021). The interplay of SARS-CoV-2 and Clostridioides difficile infection. Future Microbiol.

[7] Maslennikov R, Ivashkin V, Ufimtseva A, Poluektova E, Ulyanin A (2022). Clostridioides difficile co-infection in patients with COVID-19. Future Microbiol.

[8] Sandhu A, Tillotson G, Polistico J (2020). Clostridioides difficile in COVID-19 patients, Detroit, Michigan, USA, March-April 2020. Emerg Infect Dis.

[9] Sheikh AA, Sheikh AB, Shah I, Khair AH, Javed N, Shekhar R (2021). COVID-19 and fulminant Clostridium difficile colitis co-infection. Eur J Case Rep Intern Med.

[10] Hernández-García R, Garza-González E, Miller M, Arteaga-Muller G, Galván-de los Santos AM, Camacho-Ortiz A (2015). Application of the ATLAS score for evaluating the severity of Clostridium difficile infection in teaching hospitals in Mexico. Braz J Infect Dis.

[11] Azimirad M, Noori M, Raeisi H (2021). How does COVID-19 pandemic impact on incidence of Clostridioides difficile infection and exacerbation of its gastrointestinal symptoms?. Front Med (Lausanne).

[12] Fernandez-Cotarelo MJ, Nagy-Agren SE, Smolkin ME, Jimenez-Diez-Canseco L, Perez-Pomata MT, Shenal BV, Warren CA (2019). Functional and cognitive status in Clostridium difficile infection in the hospitalized elderly: a retrospective study of two sites. J Gen Intern Med.

[13] Gotoh K, Sakaguchi Y, Kato H (2022). Fecal microbiota transplantation as therapy for recurrent Clostridioides difficile infection is associated with amelioration of delirium and accompanied by changes in fecal microbiota and the metabolome. Anaerobe.

[14] Matheson JT, Holsinger RM (2023). The role of fecal microbiota transplantation in the treatment of neurodegenerative diseases: a review. Int J Mol Sci.

[15] Jorgensen SC, Tse CL, Burry L, Dresser LD (2020). Baricitinib: a review of pharmacology, safety, and emerging clinical experience in COVID-19. Pharmacotherapy.

